# Heterogeneity of circulating CD8 T-cells specific to islet, neo-antigen and virus in patients with type 1 diabetes mellitus

**DOI:** 10.1371/journal.pone.0200818

**Published:** 2018-08-08

**Authors:** Sandra Laban, Jessica S. Suwandi, Vincent van Unen, Jos Pool, Joris Wesselius, Thomas Höllt, Nicola Pezzotti, Anna Vilanova, Boudewijn P. F. Lelieveldt, Bart O. Roep

**Affiliations:** 1 Department of Immunohematology and Blood Transfusion, Leiden University Medical Center, Leiden, the Netherlands; 2 Computational Biology Center, Leiden University Medical Center, Leiden, the Netherlands; 3 Computer Graphics and Visualization, Delft University of Technology, Delft, the Netherlands; 4 Department of LKEB Radiology, Leiden University Medical Center, Leiden, the Netherlands; 5 Department of Diabetes Immunology, Diabetes & Metabolism Research Institute at the Beckman Research Institute, City of Hope, Duarte, California, United States of America; Universita degli Studi di Palermo, ITALY

## Abstract

Auto-reactive CD8 T-cells play an important role in the destruction of pancreatic β-cells resulting in type 1 diabetes (T1D). However, the phenotype of these auto-reactive cytolytic CD8 T-cells has not yet been extensively described. We used high-dimensional mass cytometry to phenotype autoantigen- (pre-proinsulin), neoantigen- (insulin-DRIP) and virus- (cytomegalovirus) reactive CD8 T-cells in peripheral blood mononuclear cells (PBMCs) of T1D patients. A panel of 33 monoclonal antibodies was designed to further characterise these cells at the single-cell level. HLA-A2 class I tetramers were used for the detection of antigen-specific CD8 T-cells. Using a novel Hierarchical Stochastic Neighbor Embedding (HSNE) tool (implemented in Cytosplore), we identified 42 clusters within the CD8 T-cell compartment of three T1D patients and revealed profound heterogeneity between individuals, as each patient displayed a distinct cluster distribution. Single-cell analysis of pre-proinsulin, insulin-DRIP and cytomegalovirus-specific CD8 T-cells showed that the detected specificities were heterogeneous between and within patients. These findings emphasize the challenge to define the obscure nature of auto-reactive CD8 T-cells.

## Introduction

A hallmark of autoimmune type 1 diabetes (T1D) is the destruction of pancreatic β-cells. Several studies have demonstrated the critical role of auto-reactive CD8 T-cells in the disease pathogenesis [1–4]. β cell-specific CD8 T-cells are present in the blood of T1D patients, although in very low frequencies. Several HLA-A2 restricted islet epitopes have been associated with T1D, including pre-proinsulin (PPI), GAD65, IA-2, IGRP and ZnT8 [5]. We recently discovered a novel nonconventional self-epitope derived from an alternative open reading frame of insulin mRNA [6]. CD8 T-cells directed against this defective ribosomal product (DRIP) destroyed human β-cells *in vitro*.

The question remains whether auto-reactive CD8 T-cells possess certain phenotypic characteristics distinguishing these from CD8 T-cells of other specificities, such as virus-specific CD8 T-cells. Several lines of evidence suggest that auto-reactive T-cells display features that are distinct from non-self-reactive T-cells, which may have contributed to their escape from central tolerance. These differences include low TCR binding affinity of their epitopes in HLA context, low TCR avidity, impaired formation of immunological synapse with antigen presenting cells and elevated Kv1.3 potassium channel expression [7–9]. CD8 T-cells reactive to neo-antigens are supposedly unaffected by thymic elimination if not expressed in the thymus, and may thus look similar to T-cells against pathogens. Improved definition of auto-reactive T-cell phenotype could aid in early detection of disease, monitoring of disease progression or even provide applications for immune intervention.

Therefore, the aim of this study was to utilize a detection method using HLA-A2 class I tetramers specific for the epitopes PPI_15-24_ (auto-reactive), INS-DRIP_1-9_ (neo-reactive) and CMV-pp65_495-503_ (virus-reactive) to detect peptide-specific CD8 T-cells in mass cytometry and to assess whether these specific CD8 T-cells in T1D patients display a distinct phenotype. To this end, we applied high-dimensional mass cytometry by time of flight (CyTOF) to analyse 33 immune markers simultaneously at the single-cell resolution. With a novel hierarchical HSNE [10, 11], incorporating t-SNE and HSNE, 42 clusters were distinguished within the total CD8 T-cell compartment. Single-cell analysis gave in depth information of circulating PPI, INS-DRIP and CMV CD8 T-cell phenotypes detected in T1D patients. Dissection of the CD8 T-cell compartment unraveled heterogeneity between patients as well as profound heterogeneity of the antigen-specific CD8 T-cell pool within an individual, highlighting the complexity to identify a biomarker for islet-specific CD8 T-cells in T1D patients.

## Materials and method

### Human blood samples

Blood samples from T1D patients were collected in 10 ml sodium heparin tubes. Between 10–50 ml volume of blood was collected per patient. Patients signed informed consent and this study was approved by the medical-ethical committee of the LUMC. The clinical characteristics of these patients are shown in S1 Table.

### Antibody staining panel

The panel of metal conjugated antibodies was designed using a strategy that provides maximum signal detection with a minimal background. A 33 marker panel (Table 1) which can potentially discriminate PPI-, INS-DRIP- and CMV-reactive cells was designed including markers that distinguish cell lineages, naïve from memory and resting from activated cells. Metal conjugated antibodies were either purchased pre-conjugated or self-conjugated using purified antibodies (lacking carrier proteins). The antibodies were labelled 100 μg at a time with the desired lanthanide using a MaxPar X8 antibody labeling kit (Fluidigm, Sciences, Toronto, Canada). After conjugation, antibodies were diluted to 200 μl in Candor PBS Antibody Stabilization Buffer (Candor Bioscience GmbH, Wangen, Germany) and stored at 4°C. Performance check of MaxParMetal Conjugated Antibodies was done by adding antibody to one comp-beads (Affymetrix, eBiosciences San Diego, CA, USA) followed by acquiring beads on CyTOF2 (Fluidigm Sciences, USA). The staining concentration of each marker was determined by titration on a mix of non-stimulated and PHA stimulated PBMCs.

**Table 1:** Staining panel for surface markers.

Marker	Metal	Clone	Dilution
Anti-PE	156Gd	PE001	1:50
CD3	170Er	UCHT1	1:100
CD4*	146Nd	RPA-T4	1:200
CD7	153Eu	CD7-6B7	1:100
CD8a*	145 Nd	RPA-T8	1:50
CD16	148Nd	3G8	1:100
CD20*	163Dy	2H7	1:200
CD25	149Sm	2A3	1:100
CD27	167Er	O323	1:100
CD28*	171Yb	CD28.2	1:100
CD38	172Yb	HIT2	1:200
CD45	89Y	HI30	1:100
CD45RA	169Tm	HI100	1:100
CD45RO*	173Yb	UCHL1	1:100
CD49b*	176Yb	P1e6c5	1:100
CD69	144Nd	FN50	1:50
CD103*	155Gd	Ber-ACT8	1:50
CD107 (LAMP)*	143Nd	H4A3	1:50
CD122*	158Gd	TU27	1:50
CD126 (IL6R)*	154Sm	UV4	1:40
CD127	165Ho	AO19D5	1:200
CD152 (CTLA4)*	166Er	14D3	1:40
CD161	164Dy	HP-3G10	1:100
CD196 (CCR6)	141Pr	G034E3	1:100
CD197 (CCR7)	159Tb	G043H7	1:100
CD223 (LAG)	150Nd	874501	1:40
CD278 (ICOS)	151Eu	DX29	1:50
CD279 (PD-1)	175Lu	EH 12.2H7	1:100
CD335 (NKp46)*	174Yb	9E2	1:50
CD336 (Nkp44)*	147Sm	P44-8	1:50
CD357 (GITR)*	142Nd	621	1:40
HLA-DR*	168Er	L243	1:200
TCRgd	152Sm	11F2	1:50
KLRG-1*	160Gd	REA261	1:50

*self-conjugated

### Validation of tetramer staining

To facilitate staining of the specific HLA-A2-restricted CD8 T-cells, pre-proinsulin PPI_15-24_ *(ALWGPDPAAA)*, INS-DRIP_1-9_ *(MLYQHLLPL)*, and virus CMV-pp65_495-503_ *(NLVPMVATV)* HLA-A2^+^ tetramers (Tm) fluorescently labelled with phycoerythrin (PE) were generated as previously described [12]. These tetramers have been extensively tested and validated for FACS analysis in earlier studies [5, 7, 13]. To validate the detection of antigen-specific CD8 T-cells in CyTOF2, clones with specificity against the selected tetramers were spiked in HLA-A2 negative PBMC at a frequency of 1% (S1 Fig). Samples were labelled with PE-labelled tetramers (1ng/μl) at room temperature for 45 minutes. After washing the cells in cold PBS containing 0.05% BSA, cells were kept at 4°C. Samples were split in two to compare the tetramer staining in FACS and CyTOF2. FACS samples were stained additionally with CD8-FITC antibody for 20 minutes and measured on the FACS Calibur (Becton Dickinson). CyTOF samples were washed twice with Maxpar Cell Staining Buffer (CSB) (Fluidigm Sciences, USA) and stained further as described in the next paragraph. As negative control, non-spiked samples were stained using the same method.

### Isolation and staining of PBMC- derived CD8 T-cells for mass cytometry

PBMCs from three HLA-typed T1D patients were isolated from blood using Ficoll-Plaque density gradient centrifugation. PBMCs were cryopreserved in 50% IMDM, 40% fetal calf serum and 10% DMSO and stored in vials of 10 to 25x10^6^ cells in 1 ml. Blood samples were processed within 24 hours after collection. PBMCs were stored for 6 to 30 months before analysis and thawed in 50% fetal calf serum and 50% IMDM. Thereafter, cells were spun for 5 minutes at 1600 RPM and recovered for 30 minutes in IMDM supplemented with 10% human serum to minimize cell death. The viability of the samples after freeze and thaw was above 98%. To minimize inter-assay variation caused by measurement time, samples above 2x10^6^ per condition were enriched for CD8 T-cells by depleting CD14, CD19 and CD4 using Macs microbeads (Miltenyi Biotec, Bergisch Gladbach, Germany) according to the supplier’s protocol. Depleting did not affect tetramer staining, CD8 T-cells remained untouched. Depleted PBMCs were counted and 2.5x10^6^ cells per sample were washed in PBS containing 0.5% BSA, 0.02% Sodium azide (FACS buffer) prior to staining with the desired specific PE-labelled tetramers (1ng/μl) at room temperature for 45 minutes. After washing the cells once in cold PBS containing 0.05% BSA, cells were kept at 4°C and washed twice with Maxpar Cell Staining Buffer (CSB), (Fluidigm Sciences, USA) before adding a cell ID Interchalator-^103^Rh (Fluidigm Sciences, USA; 1μM in CSB) for 20 min. After washing with CSB, non-specific binding of Fc receptor expressing cells was blocked (Human TruStain FcX, Biolegend) for 15 min and thereafter cells were labelled with anti-PE ^156^Gd to retrieve the PE-labelled tetramer staining (45 min on ice). A mixture of antibodies (listed in Table 1) diluted in CSB was added for 45 min. After washing the cells three times in CSB, a cell ID Interchalator ^191^Ir ^193^Ir (Fluidigm Sciences, USA) 125 nM diluted in Maxpar Fix and Perm buffer (Fluidigm Sciences, USA) was added overnight up to 48 hours. Cells were stored at 4°C before being tested on CyTOF2.

### Data acquisition

Before being tested on CyTOF2, cells were washed twice with CSB and once in distilled water before dilution to the appropriate concentration (0.5x10^6^ cells/ml) to achieve a proper acquisition rate on the CyTOF2. To each sample 1/10 volume of Calibration Beads, EQ(TM) Four Element (Fluidigm Sciences, USA) was added to be able to normalize data sets. CyTOF2 data were acquired and analysed on the fly, using dual-count mode and noise-reduction. All other settings were either default settings or optimized with tuning solution as instructed by Fluidigm Sciences. After data acquisition, the mass of the bead signal (EQ passport P13H2302) was used to normalize the data. The number of events acquired per sample is shown in S2 Table.

### Data analysis

We discriminated live, single cells using DNA stains and event length. Furthermore, cells were gated based on CD4^-^, TCRgd^-^, CD3^+^ and CD8^+^ and compensation beads were excluded. The population of CD8 T-cells was gated based on tetramer expression (tetramer positive or negative) determined for each epitope. FCS files were exported from Cytobank [14]. Data were transformed using hyperbolic ArcSin with cofactor 5. Anti-PE expression was excluded in all further clustering analysis. Dimensionality reduction techniques t-SNE and HSNE [11] were used to analyse the high-resolution mass cytometry datasets. HSNE was used to dissect the composition of the CD8 T-cells with a full scale resolution. HSNE removes the scalability limit of conventional t-SNE analysis and is therefore highly suitable for the analysis of massive high-dimensional datasets [10]. t-SNE is carried out using the A-tSNE implementation of t-SNE [15], using the Barnes-Hut optimization of t-SNE [16] for computational efficiency. t-SNE and HSNE were performed using the recently developed tool ‘Cytosplore’ [17]. Each single cell analysed in Cytosplore obtains a unique tag number, which facilitates cell tracing in the embedding. Density-based clustering with a Gaussian-mean-shift method distributed PPI-, INS-DRIP-, CMV-reactive cells and tetramer negative CD8 T-cells in distinct clusters. Clustering was based on the expression profile of the markers listed in Table 1 excluding anti-PE tetramer. A one-way hierarchical clustering heat map was generated in Cytosplore including the variations in marker expression in each cluster. t-SNE analyses were performed with random down sampling of tetramer negative (150,000), together with tetramer positive CD8 T-cells not down sampled, to visualize marker expression of each individual cell in the embedding focusing on the tetramer positive fraction with a high single-cell resolution. Snapshots of the embedding display the selected marker expression of the total CD8 T-cell compartment per patient. Furthermore, t-SNE x and y coordinates of CD8 T-cells of each individual sample were used for Jensen-Shannon (JS) analysis. JS-plots were generated using Matlab version R2016a (MATLAB6.1, The MathWorks Inc., Natick, MA, 2000). Single-cell two-way hierarchical clustering heat maps were generated based on intensity value of 33 markers using R software (R package, version 1.0.153). Packages ‘flowcore’, ‘ggplot2’, ‘gplots’ and ‘heatmap.2’ were used to assist clustering and heat map drawing. Tetramer negative CD8 T-cells of patient 1 and 3 were down sampled to a similar number of total PPI-, INS-DRIP-, and CMV-reactive cells per patient. In light of the very high frequency of CMV-reactive cells in patient 2, these were down sampled to the total of PPI- and INS-DRIP-reactive cells first, followed by down sampling of tetramer negative cells to a similar number of total PPI-, INS-DRIP-, and CMV-reactive cells.

## Results

### Detection of PPI-, INS-DRIP- and CMV-reactive cells using CyTOF

PPI-, INS-DRIP- and CMV-reactive cells were detected using HLA class I tetramers in PBMCs of three T1D patients and co-stained with a 33 surface marker panel (S1A Fig). To confirm the detection of epitope-specific cells, we spiked a CD8 T-cell clone with reactivity against PPI_15-24_, INS-DRIP_1–9_ or CMV-pp65_495-503_ in PBMCs of a healthy HLA-A2 negative patient followed by staining and acquiring with the mass cytometer (S1B Fig). The detection of specific tetramer positive CD8 T-cells spiked in PBMCs of HLA-A2 negative PBMCs was confirmed using regular flow cytometry. Auto-reactive CD8 T-cells against PPI_15-24_ have been detected in recent onset patients with frequencies around 0.03% [5]. We recently showed neo-reactive-specific CD8 T-cells against INS-DRIP_1–9_ detected at frequencies as low as 0.03% [6]. Using mass cytometry, we detected PPI_15-24-_specific CD8 T-cells with frequencies between 0.014–0.078% and INS-DRIP_1–9_-specific cells with frequencies between 0.028–0.049%, both comparable to earlier observed increased frequencies in T1D patients. CMV-pp65_495-503_*-*specific CD8 T-cells appeared in a broader range of frequencies 0.007–5.960% (S1C Fig).

### Composition of the CD8 T-cell compartment in an HSNE and t-SNE embedding

**Fig 1:**
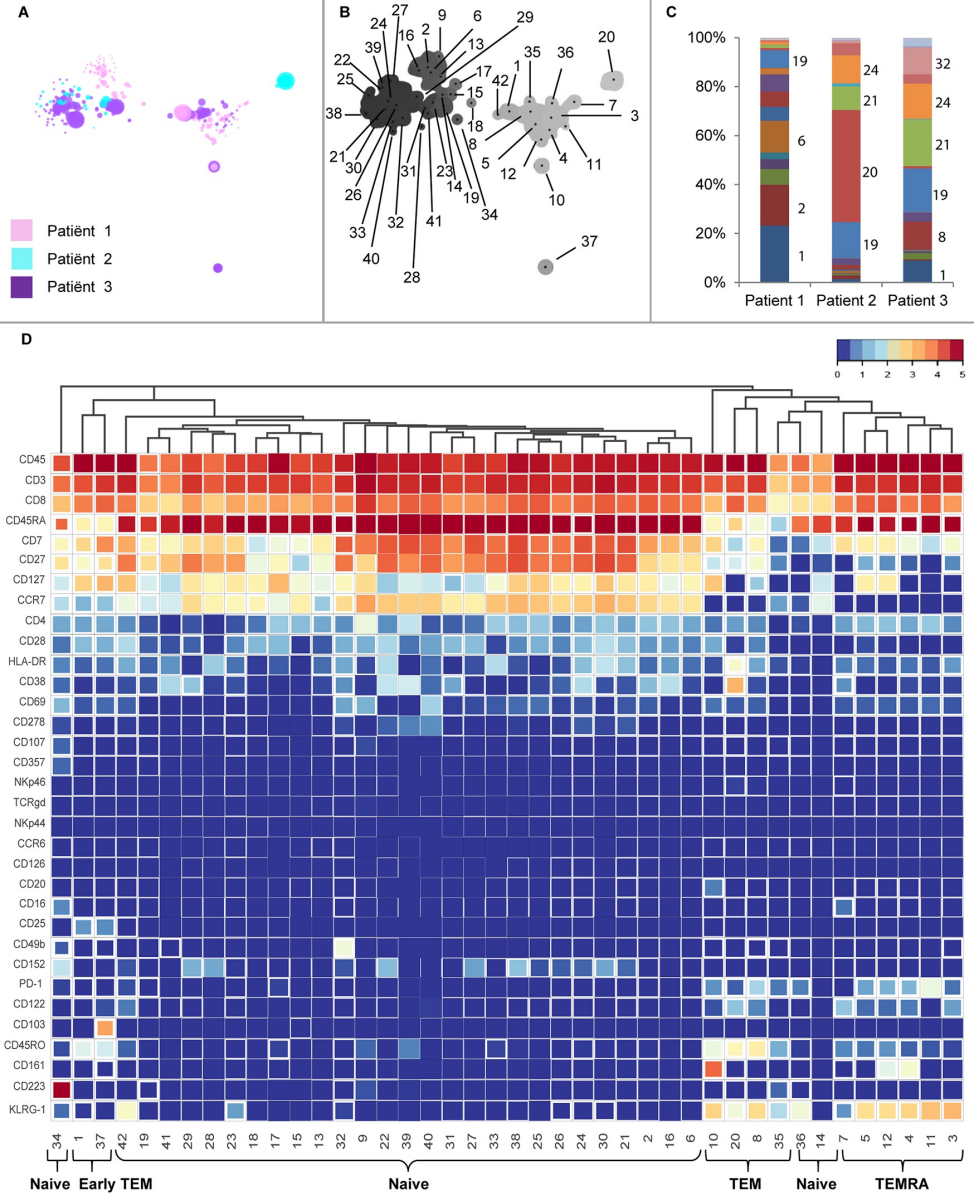
Dissection of the CD8 compartment of three T1D patients. a) Overview HSNE of CD8 compartment, clustering was based on marker expression of 33 markers after hyperbolic Arcsin5 transformation. b) Density map including cluster number. A sigma value of 7 resulted in 42 clusters. c) Distribution of clusters per patient. d) Heat map view including variation in marker expression within a cluster (box fill–smaller box depicts higher variation).

To obtain a global overview of the marker expression in the total CD8 T-cell compartment, a novel dimensionality reduction technique HSNE was implemented [10]. In conventional t-SNE analysis, large numbers of cells lead to an overcrowding of the analysis, hence the need for down sampling of the dataset. HSNE is a t-SNE based tool with the ability to process large datasets, which facilitates the identification of low frequency cell populations. A total of 705,402 cells including PPI, INS-DRIP and CMV-specific tetramer positive and negative CD8 T-cells of three T1D patients were analysed. An HSNE embedding was generated based on 33 markers listed in Table 1, excluding the tetramer staining intensity. The embedding at the overview level revealed landmarks (representative data points) of cell populations with distinct marker expression patterns (Fig 1A). Using the Gaussian-mean-shift method, 42 clusters were identified representing the CD8 T-cell compartment and preserving subtle differences in the marker expression. Each cluster was reflected in the landmarks at the overview level (Fig 1B). Interestingly, each patient showed a distinct cluster distribution in cell frequencies (Fig 1C and S2 Fig). In a heat map visualization of these 42 clusters, various phenotypes could be distinguished. Firstly, clusters could be divided into effector memory cells (TEM) expressing CD45RA^-^CD45RO^+^CCR7^-^ (cluster 8, 10, 20, 35), early effector memory cells (early TEM) expressing CD45RA^lo^CD45RO^lo^CCR7^lo^ (cluster 1, 37), terminally differentiated (TEMRA) cells expressing CD45RA^+^CD45RO^+^CCR7^-^ (cluster 3, 4, 5, 7, 11, 12) and naïve cells expressing CD45RA^+^CD45RO^-^CCR7^+^ (cluster 2, 6, 9, 13–19, 21–34, 36, 38–42) (Fig 1D). Additional diversity was seen within each category. For example, within the clusters described as a TEM phenotype, CD161 was expressed in cluster 10, whereas CD38 was expressed in cluster 20. Cells with an early TEM phenotype (cluster 1 and 37) were separated by the expression of CD103 (cluster 37). Within the naïve clusters, CD223 expression was present exclusively on cells in cluster 34. The distinctiveness of these clusters is reflected at the overview level, where they can be identified as separate landmarks in the embedding (Fig 1B).

**Fig 2:**
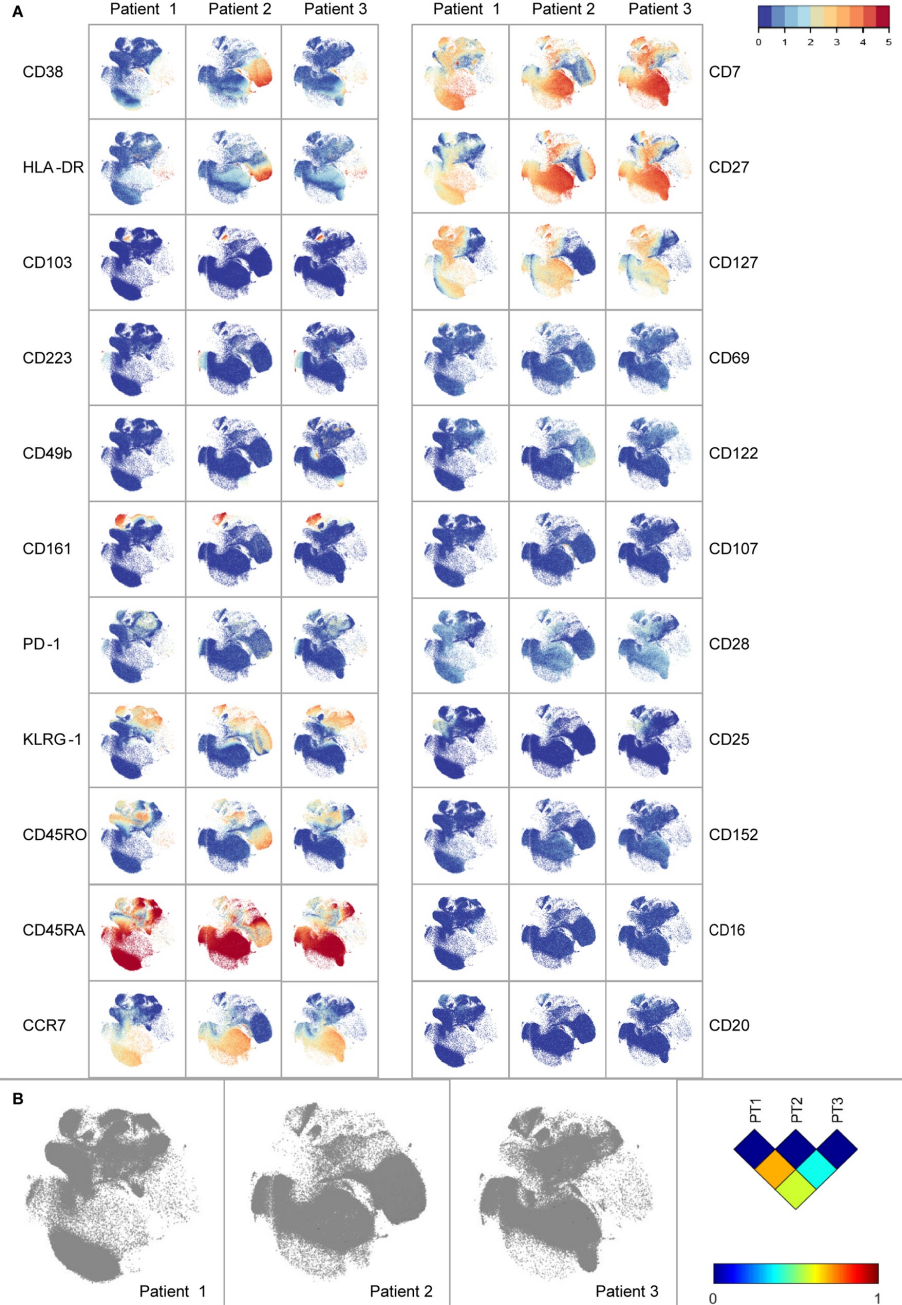
Marker expression visualization. a) t-SNE of CD8 compartment of three T1D patients. Markers CD8, CD3 and TCRgd used for gating, as well as the negative markers NKp44 and NKp46 are not displayed. b) Jensen-Shannon divergence plots of t-SNE maps comparing three patients. Higher values indicate more dissimilarity between a pair of t-SNE maps.

In order to visualize global marker expression profiles at the single-cell level, CD8 T-cells were analysed in a single t-SNE embedding (Fig 2A). CD8 T-cells expressing CD161, CD103 and CD223 were present in all three patients. On the other hand, several markers were expressed in a patient-specific manner: CD25 was mainly expressed on CD8 T-cells from patient 1 and 3, CD38 and HLA-DR were almost exclusively expressed on CD8 T-cells from patient 2, whereas CD49b was only expressed on cells from patient 3. t-SNE analysis per patient was performed to visualize different embedding (S3 Fig). To quantify the previously observed dissimilarities between the CD8 compartments of the three T1D patients, the Jensen-Shannon divergence was calculated between the t-SNE maps (Fig 2B). A higher JS-divergence value indicated more dissimilarity in distribution of cells between a pair of t-SNE maps. Each patient had a distinct composition of CD8 T-cells, with a most pronounced difference between patients 1 and 2 with a JS-divergence of 0.6938 (S4B Fig).

### Single-cell heat map per patient displayed heterogeneity within PPI-, INS-DRIP- and CMV-reactive cells

**Fig 3:**
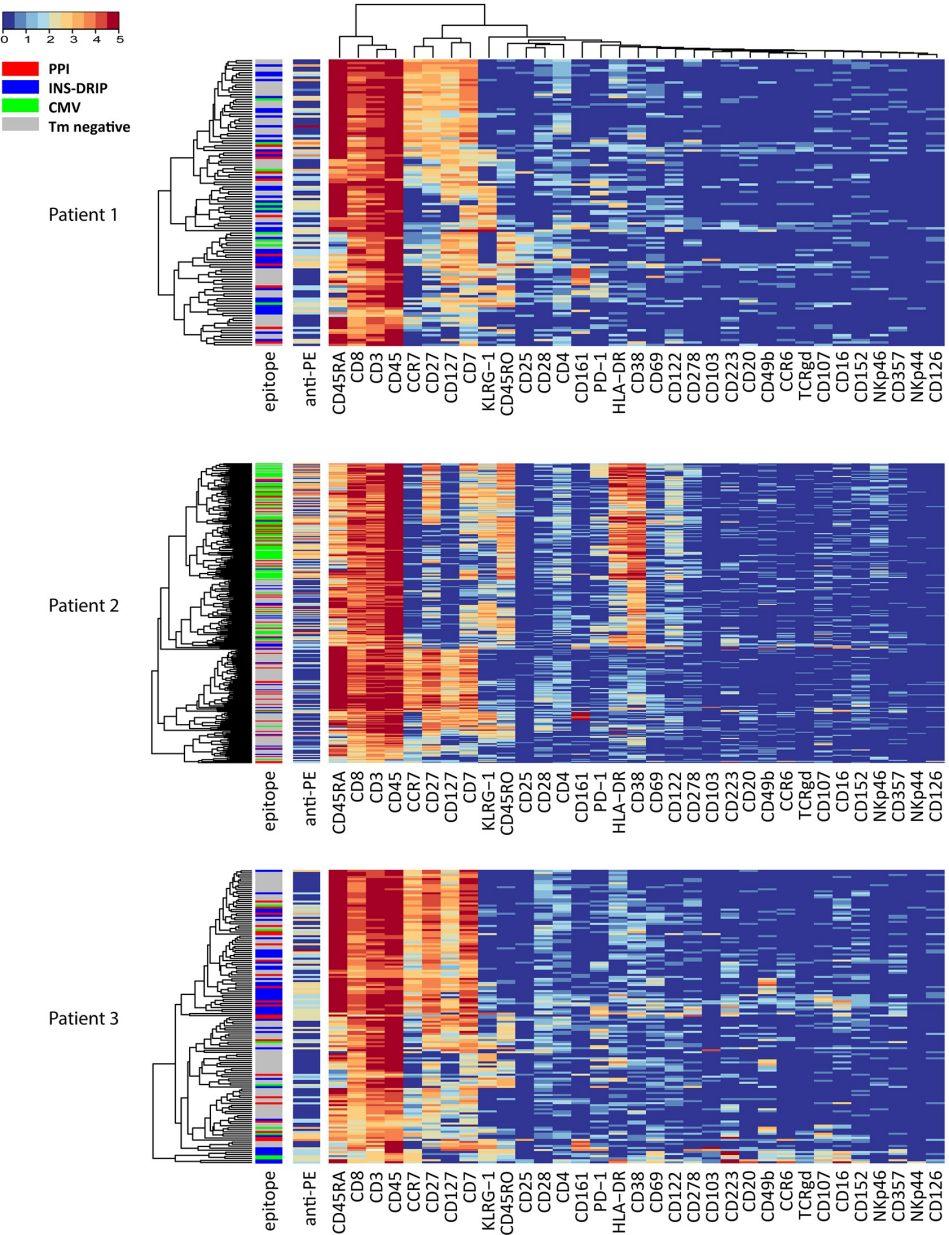
Single-cell heat map of CD8 T-cells of three T1D patients. The first sidebar displays the specificity (red = PPI, blue = DRIP, green = CMV, grey = tetramer negative), the second bar is depicting the tetramer signal (not taken into account for clustering).

Next, we focussed on the CD8 T-cell diversity of the PPI-, INS-DRIP- and CMV-reactive cells, using tetramer negative cells as a control, which were randomly down sampled to equal numbers of tetramer positive cells. A single-cell heat map of CD8 T-cells of all patients together clearly showed patient driven clustering (S4A Fig) in line with our previous findings. Therefore, we generated single-cell heat maps per patient to compare tetramer negative CD8 T-cells with PPI-, INS-DRIP- and CMV-reactive cells within an individual (Fig 3). The heat map of patient 1 is divided in two main clusters with CD45RA and CD45RO expression profiles being the main discriminators. On a second level, subgroups are divided by the expression of CCR7 and CD27. Within these major groups, subgroups were present based on KLRG-1 and CD161 expression profiles. PPI-, INS-DRIP- and CMV-reactive cells were intermixed with tetramer negative cells and not clustered together. On the contrary, each single cell displayed a more unique phenotype, pointing out heterogeneity of peptide-specific responses. For patient 2, CD38, CD45RO and HLA-DR determined the first branch of clustering. Virus-reactive cells shared a common phenotype expressing CD38, CD45RO and HLA-DR, but were lacking the expression of CD127 and CCR7. Within this cluster, heterogeneity was observed based on the marker expression of KLRG-1, CD27 and CD7. The islet-specific PPI- and INS-DRIP-reactive cells displayed a more diverse distribution pointing at unique phenotypes in patient 2. First level of clustering in case of patient 3 was based on the expression of CD45RA, CCR7 and CD127. The PPI islet-specific CD8 T-cells were distributed over these clusters, pointing again at heterogeneity. Remarkably, INS-DRIP cells of patient 3 were predominantly clustering separate from CD8 tetramer negative cells in the CD45RO^-^CD45RA^+^CCR7^+^CD127^+^ cluster and a second group of INS-DRIP-specific clusters being CD45RO^+^, CD69^+^, CD16^+^, CCR6^+^ and CD223^+^.

**Fig 4:**
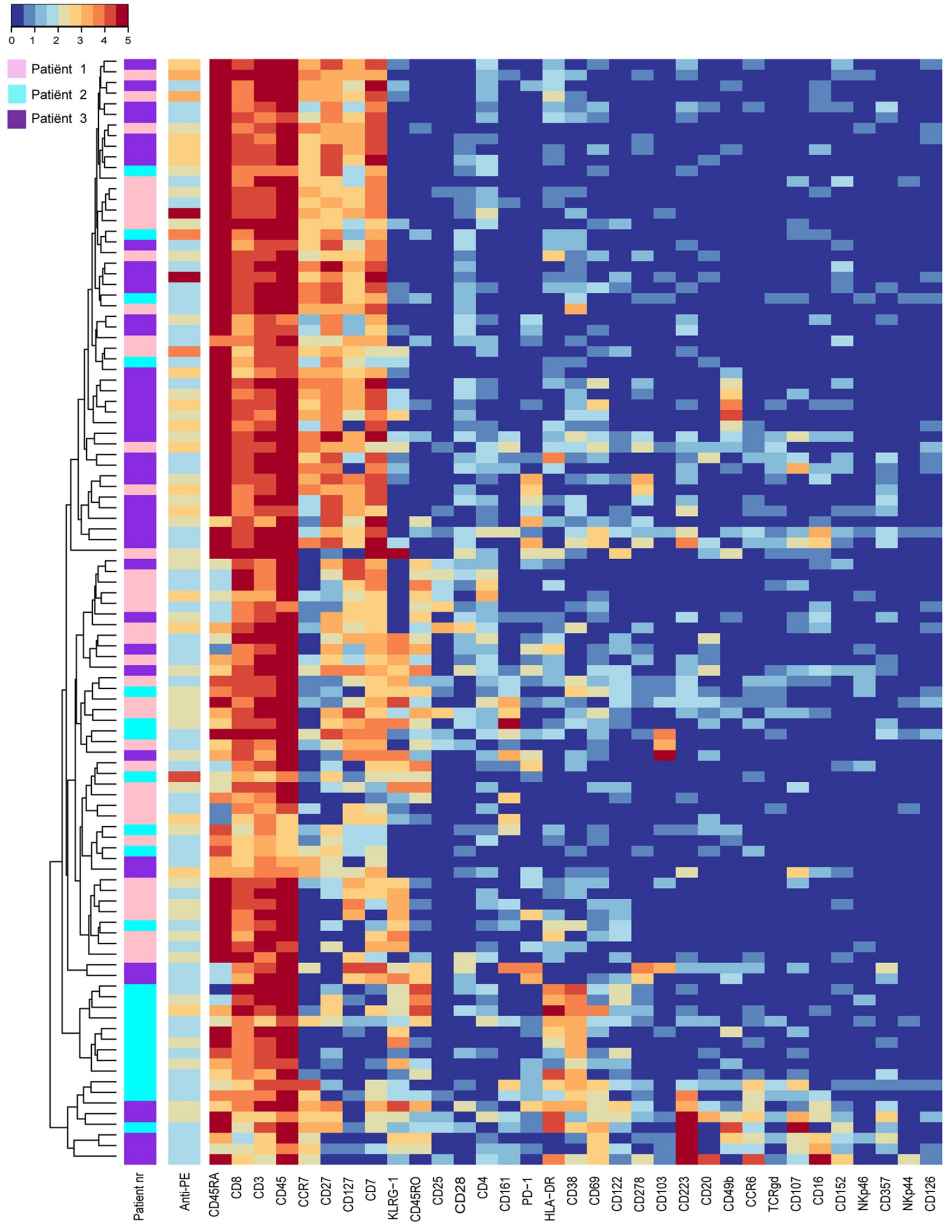
Single-cell heat map of INS-DRIP-specific CD8 T-cells. The first sidebar displays patient origin (pink = patient 1, light-blue = patient 2, purple = patient 3), the second bar is depicting the tetramer signal.

INS-DRIP-specific CD8 T-cells were detected in similar frequencies in the three patients. To assess whether these INS-DRIP-specific CD8 T-cells from different patients have similar features, cells were analysed in a single-cell heat map (Fig 4). Naïve INS-DRIP-specific CD8 T-cells were present in all three patients and represented a population expressing CD45RA, CCR7, CD127, CD27 and CD7. INS-DRIP-specific CD8 T-cells derived from patient 3 showed variation in this population by expressing CD49b, PD-1 and CD223 in different clusters. Memory INS-DRIP-specific CD8 T-cells were more heterogeneous with variable KLRG-1 expression and expression of HLA-DR and CD38 in patient 2. Another cluster expressing CD45RA and KLRG-1 and lacking CCR7, showing an exhausted phenotype, was mainly represented by cells from patient 1.

In summary, within the CD8 T-cell compartment of all three patients we identified TEM, TEMRA, early TEM and naïve cells, as well as populations with diverse marker expression patterns such as CD161, CD223 and CD103. Additionally, several markers were only expressed by cells derived from a single patient, such as CD38 and HLA-DR in case of patient 2, which is in line with the distinct cluster distribution pattern and the high JS-divergence between patients (paragraph 1). A stratified approach analysing PPI, INS-DRIP and CMV-specific cells within one patient showed that these CD8 T-cells did not share a single marker expression pattern, but were highly heterogeneous and mostly intermixed with tetramer negative cells. However, within INS-DRIP-specific cells, additional variety of antigen-specific CD8 T-cells was observed.

## Discussion

Islet-specific CD8 T-cells are key players involved in the development of T1D and occur in both the circulation and insulitic lesions [1, 5]. The phenotype of these cells has not yet been described extensively. In this study we combined antigen-specific HILA-class I tetramer staining with a broad surface marker panel to characterise circulating antigen-specific CD8 T-cells. The selected patients were variable in age of onset and disease duration, allowing evaluation of patients in different disease states in high resolution regarding CD8 T-cell composition and specificity. For this purpose, a stratified approach was used in the single-cell analysis. In addition to these three patients, a series of patients was analysed with differing combination of markers. The latter precludes inclusion in the same database, since samples with a different combination of labels cannot be compared in the same analysis. The experimental design using cryopreserved human PBMCs was elected for its potential to larger scale and longitudinal application and to minimize inter-assay variability. However, effects of cryopreservation on the marker expression should be taken into account. The expression of CD4, CD8, CD45RA, CD45RO, CD38, CD28 and HLA-DR has been shown to be stable after cryopreservation [18].

CD8 T-cells specific for the auto-antigen PPI and the recently described neo-antigen INS-DRIP, as well as CMV-reactive cells, were detected in all three T1D patients in order to unravel phenotypical differences between auto-, neo- and virus-reactive cells. We detected low frequencies of PPI- and INS-DRIP-specific CD8 T-cells similar to previously reported data [5, 6]. Additionally, the tetramer intensity of the PPI- and INS-DRIP-specific CD8 T-cells were of low intensity, which is in line with findings that auto-reactive T-cells have a low-affinity binding TCR [7, 13]. This may explain their presence in the periphery, as low affinity might enable escape from negative thymic selection. The phenotypical composition of CD8 T-cells in the three T1D patients was highly variable. Remarkably, within patients the specific PPI, INS-DRIP and CMV CD8 T-cells were not distinguishable based on the selected markers, but were heterogeneous.

HSNE dissection of the CD8 T-cell compartment unraveled a wide variety of phenotypes containing the well described TEM, early TEM, TEMRA and naïve cells, and further variety within these clusters. Focusing on markers forming distinct clusters, we find TEM cells in cluster 10 expressing CD161, a tissue homing marker for CD8 T-cells. [19]. Furthermore, CD103 expression on a cluster of early TEM cells is described as a marker observed in tissue resident memory T-cells [20]. CD223 (Lag-3), which is expressed on a cluster of naïve cells, is a cell surface molecule with diverse biologic effects on T-cell function and helps to maintain CD8 T-cells in a tolerogenic state [21]. Yet, CD161, CD103 and CD223 did not distinguish CD8 T-cells specific for PPI, INS-DRIP or CMV. Interestingly, the expression of certain markers was patient-dependent. The interleukin-2 receptor alpha subunit CD25 is mainly present on CD8 T-cells from patient 1 and 3. Furthermore, high numbers of CD8 T-cells expressing activation markers CD38 and HLA-DR are observed in patient 2, these are mainly CMV-specific cells. High expression of CD38 and HLA-DR might be caused by an active CMV infection which is in accordance with a relatively high percentage of detected CMV-specific CD8 T-cells. Finally, CD49b expression was found on a cluster of cells only in patient 3. CD49b is an integrin alpha 2 subunit reported to be one of the six very late antigens on activated T-cells [22]. Heterogeneity of CD8 T-cells between individuals greatly influenced the HSNE and t-SNE based analysis strategies. It affected our aim to find biomarkers for islet-specific cells, since we could not analyse and compare the data of all three T1D patients together.

In our search to identify immune signatures for islet-specific cells, we compared CD8 T-cells with PPI-, INS-DRIP- and CMV-reactive cells at the single-cell level per patient. We visualized the complexity of CD8 T-cell subpopulations, but could not reveal a distinct subpopulation containing only islet-specific cells. Peptide-specific cells of PPI-, INS-DRIP- and CMV-reactive cells were however present throughout several of these subpopulations. The markers CD20, CD126, CD152, CD357, NKp44 and NKp46 did not add to differentiation of CD8 T-cells or their specificities, even though these have previously been reported to be expressed on T-cell subsets [23, 24].

In conclusion, the CD8 T-cell compartment is heterogeneous between patients, which also holds true for auto-antigen or virus-specific CD8 T-cells. This calls for caution when analysing combined data sets in search for biomarkers to detect rare cell populations in different individuals. Antigen-specific CD8 T-cells appear in different activation and differentiation states within a patient, while CD8 T-cells specific for different antigens (PPI, INS-DRIP or CMV) cannot be distinguished by the expression of surface markers from one another. These notions should be considered when analysing rare antigen-specific cells CD8 T-cells.

## Supporting information

S1 Fig Detection of specific CD8 T-cells. a) Schematic overview of staining peptide-specific CD8 T-cells using HLA-class I tetramers in CyTOF2. b) HLA-A2 negative PBMCs spiked with 1% PPI-specific (panel 1), 1% INS-DRIP-specific (panel 2) and 1% CMV-specific CD8 clone (panel 3). c) Dot plots and frequencies of circulating PPI, INS-DRIP and CMV reactive CD8 T-cells detected in three T1D patients.
(TIF)

S2 Fig Distribution of CD8 T-cells derived from three T1D patients per cluster. The graph shows a heterogeneous distribution of CD8 T-cells from different patients within the clusters. The order of the clusters corresponds to the heat map in Fig 1D.
(TIF)

S3 Fig t-SNE analysis of CD8 compartment. t-SNE embedding per patient, the marker expression per patient is displayed. Markers CD8, CD3 and TCRgd used for gating, as well as the negative markers NKp44 and NKp46 are not displayed.
(TIF)

S4 Fig Heat map of three T1D patients two way clustering. Left sidebar displays specificity (red = PPI, blue = INS-Drip, green = CMV, grey = CD8 tetramer negative, middle sidebar displays patient origin (pink = patient 1, light blue = patient 2, purple = patient 3), right sidebar displays the tetramer expression (anti-PE signal). b) t-SNE maps and Jensen-Shannon divergences values calculated in Matlab using SDivergenceTwoMaps.
(TIF)

S1 Table Information of patients diagnosed with T1D. (DOCX)

S2 Table Total number of events acquired per patient. (DOCX)

## References

[1] Coppieters KT, Dotta F, Amirian N, Campbell PD, Kay TW, Atkinson MA, et al Demonstration of islet-autoreactive CD8 T cells in insulitic lesions from recent onset and long-term type 1 diabetes patients. J Exp Med. 2012;209(1):51–60. 10.1084/jem.20111187 ; PubMed Central PMCID: PMCPMC3260877. 22213807 PMC3260877

[2] Tan S, Li Y, Xia J, Jin CH, Hu Z, Duinkerken G, et al Type 1 diabetes induction in humanized mice. Proceedings of the National Academy of Sciences of the United States of America. 2017;114(41):10954–9. Epub 2017/09/07. 10.1073/pnas.1710415114 ; PubMed Central PMCID: PMCPMC5642714. 28874533 PMC5642714

[3] Pinkse GG, Tysma OH, Bergen CA, Kester MG, Ossendorp F, van Veelen PA, et al Autoreactive CD8 T cells associated with beta cell destruction in type 1 diabetes. Proceedings of the National Academy of Sciences of the United States of America. 2005;102(51):18425–30. Epub 2005/12/13. 10.1073/pnas.0508621102 ; PubMed Central PMCID: PMCPMC1317949. 16339897 PMC1317949

[4] Nejentsev S, Howson JM, Walker NM, Szeszko J, Field SF, Stevens HE, et al Localization of type 1 diabetes susceptibility to the MHC class I genes HLA-B and HLA-A. Nature. 2007;450(7171):887–92. Epub 2007/11/16. 10.1038/nature06406 ; PubMed Central PMCID: PMCPMC2703779. 18004301 PMC2703779

[5] Velthuis JH, Unger WW, Abreu JR, Duinkerken G, Franken K, Peakman M, et al Simultaneous detection of circulating autoreactive CD8+ T-cells specific for different islet cell-associated epitopes using combinatorial MHC multimers. Diabetes. 2010;59(7):1721–30. Epub 2010/04/02. 10.2337/db09-1486 ; PubMed Central PMCID: PMCPMC2889772. 20357361 PMC2889772

[6] Kracht MJ, van Lummel M, Nikolic T, Joosten AM, Laban S, van der Slik AR, et al Autoimmunity against a defective ribosomal insulin gene product in type 1 diabetes. Nature medicine. 2017;23(4):501–7. Epub 2017/03/07. 10.1038/nm.4289 . 28263308

[7] Abreu JR, Martina S, Verrijn Stuart AA, Fillie YE, Franken KL, Drijfhout JW, et al CD8 T cell autoreactivity to preproinsulin epitopes with very low human leucocyte antigen class I binding affinity. Clinical and experimental immunology. 2012;170(1):57–65. Epub 2012/09/05. 10.1111/j.1365-2249.2012.04635.x ; PubMed Central PMCID: PMCPMC3444717. 22943201 PMC3444717

[8] Beeton C, Wulff H, Standifer NE, Azam P, Mullen KM, Pennington MW, et al Kv1.3 channels are a therapeutic target for T cell-mediated autoimmune diseases. Proceedings of the National Academy of Sciences of the United States of America. 2006;103(46):17414–9. 10.1073/pnas.0605136103 ; PubMed Central PMCID: PMCPMC1859943. 17088564 PMC1859943

[9] Schubert DA, Gordo S, Sabatino JJ, Jr., Vardhana S, Gagnon E, Sethi DK, et al Self-reactive human CD4 T cell clones form unusual immunological synapses. J Exp Med. 2012;209(2):335–52. 10.1084/jem.20111485 ; PubMed Central PMCID: PMCPMC3280872. 22312112 PMC3280872

[10] van Unen V, Hollt T, Pezzotti N, Li N, Reinders MJT, Eisemann E, et al Visual analysis of mass cytometry data by hierarchical stochastic neighbour embedding reveals rare cell types. Nat Commun. 2017;8(1):1740 10.1038/s41467-017-01689-9 ; PubMed Central PMCID: PMCPMC5700955. 29170529 PMC5700955

[11] Pezzotti N, Hollt T, Lelieveldt B, Eisemann E, Vilanova A. Hierarchical Stochastic Neighbor Embedding. Comput Graph Forum. 2016;35(3):21–30. 10.1111/cgf.12878 PubMed PMID: WOS:000379912300005.

[12] Altman JD, Moss PA, Goulder PJ, Barouch DH, McHeyzer-Williams MG, Bell JI, et al Phenotypic analysis of antigen-specific T lymphocytes. Science. 1996;274(5284):94–6. Epub 1996/10/04. . 8810254 10.1126/science.274.5284.94

[13] Unger WW, Velthuis J, Abreu JR, Laban S, Quinten E, Kester MG, et al Discovery of low-affinity preproinsulin epitopes and detection of autoreactive CD8 T-cells using combinatorial MHC multimers. Journal of autoimmunity. 2011;37(3):151–9. Epub 2011/06/04. 10.1016/j.jaut.2011.05.012 . 21636247

[14] Kotecha N, Krutzik PO, Irish JM. Web-based analysis and publication of flow cytometry experiments. Current protocols in cytometry. 2010;Chapter 10:Unit10.7. Epub 2010/06/26. 10.1002/0471142956.cy1017s53 ; PubMed Central PMCID: PMCPMC4208272. 20578106 PMC4208272

[15] Pezzotti N, Lelieveldt BPF, van der Maaten L, Hollt T, Eisemann E, Vilanova A. Approximated and User Steerable tSNE for Progressive Visual Analytics. Ieee T Vis Comput Gr. 2017;23(7):1739–52. 10.1109/Tvcg.2016.2570755 PubMed PMID: WOS:000402705000002. 28113434

[16] van der Maaten L. Accelerating t-SNE using Tree-Based Algorithms. J Mach Learn Res. 2014;15:3221–45. PubMed PMID: WOS:000344638800013.

[17] Hollt T, Pezzotti N, van Unen V, Koning F, Eisemann E, Lelieveldt B, et al Cytosplore: Interactive Immune Cell Phenotyping for Large Single-Cell Datasets. Comput Graph Forum. 2016;35(3):171–80. 10.1111/cgf.12893 PubMed PMID: WOS:000379912300020.

[18] Costantini A, Mancini S, Giuliodoro S, Butini L, Regnery CM, Silvestri G, et al Effects of cryopreservation on lymphocyte immunophenotype and function. Journal of immunological methods. 2003;278(1–2):145–55. . 12957403 10.1016/s0022-1759(03)00202-3

[19] Billerbeck E, Kang YH, Walker L, Lockstone H, Grafmueller S, Fleming V, et al Analysis of CD161 expression on human CD8+ T cells defines a distinct functional subset with tissue-homing properties. Proceedings of the National Academy of Sciences of the United States of America. 2010;107(7):3006–11. Epub 2010/02/06. 10.1073/pnas.0914839107 ; PubMed Central PMCID: PMCPMC2840308. 20133607 PMC2840308

[20] Shin H, Iwasaki A. Tissue-resident memory T cells. Immunological reviews. 2013;255(1):165–81. Epub 2013/08/21. 10.1111/imr.12087 ; PubMed Central PMCID: PMCPMC3748618. 23947354 PMC3748618

[21] Grosso JF, Kelleher CC, Harris TJ, Maris CH, Hipkiss EL, De Marzo A, et al LAG-3 regulates CD8+ T cell accumulation and effector function in murine self- and tumor-tolerance systems. J Clin Invest. 2007;117(11):3383–92. Epub 2007/10/13. 10.1172/JCI31184 ; PubMed Central PMCID: PMCPMC2000807. 17932562 PMC2000807

[22] Gagliani N, Magnani CF, Huber S, Gianolini ME, Pala M, Licona-Limon P, et al Coexpression of CD49b and LAG-3 identifies human and mouse T regulatory type 1 cells. Nature medicine. 2013;19(6):739–46. Epub 2013/04/30. 10.1038/nm.3179 . 23624599

[23] Schuh E, Berer K, Mulazzani M, Feil K, Meinl I, Lahm H, et al Features of Human CD3+CD20+ T Cells. J Immunol. 2016;197(4):1111–7. 10.4049/jimmunol.1600089 . 27412413

[24] Srivastava BI, Srivastava MD. Expression of natural cytotoxicity receptors NKp30, NKp44, and NKp46 mRNAs and proteins by human hematopoietic and non-hematopoietic cells. Leuk Res. 2006;30(1):37–46. 10.1016/j.leukres.2005.06.020 . 16061284

