# Supplementary figures and images for: Heterogeneity of circulating CD8 T-cells specific to islet, neo-antigen and virus in patients with type 1 diabetes mellitus
Source: PLoS One. 2018 Aug 8;13(8):e0200818. doi: 10.1371/journal.pone.0200818 (PMC6082515; doi:10.1371/journal.pone.0200818)

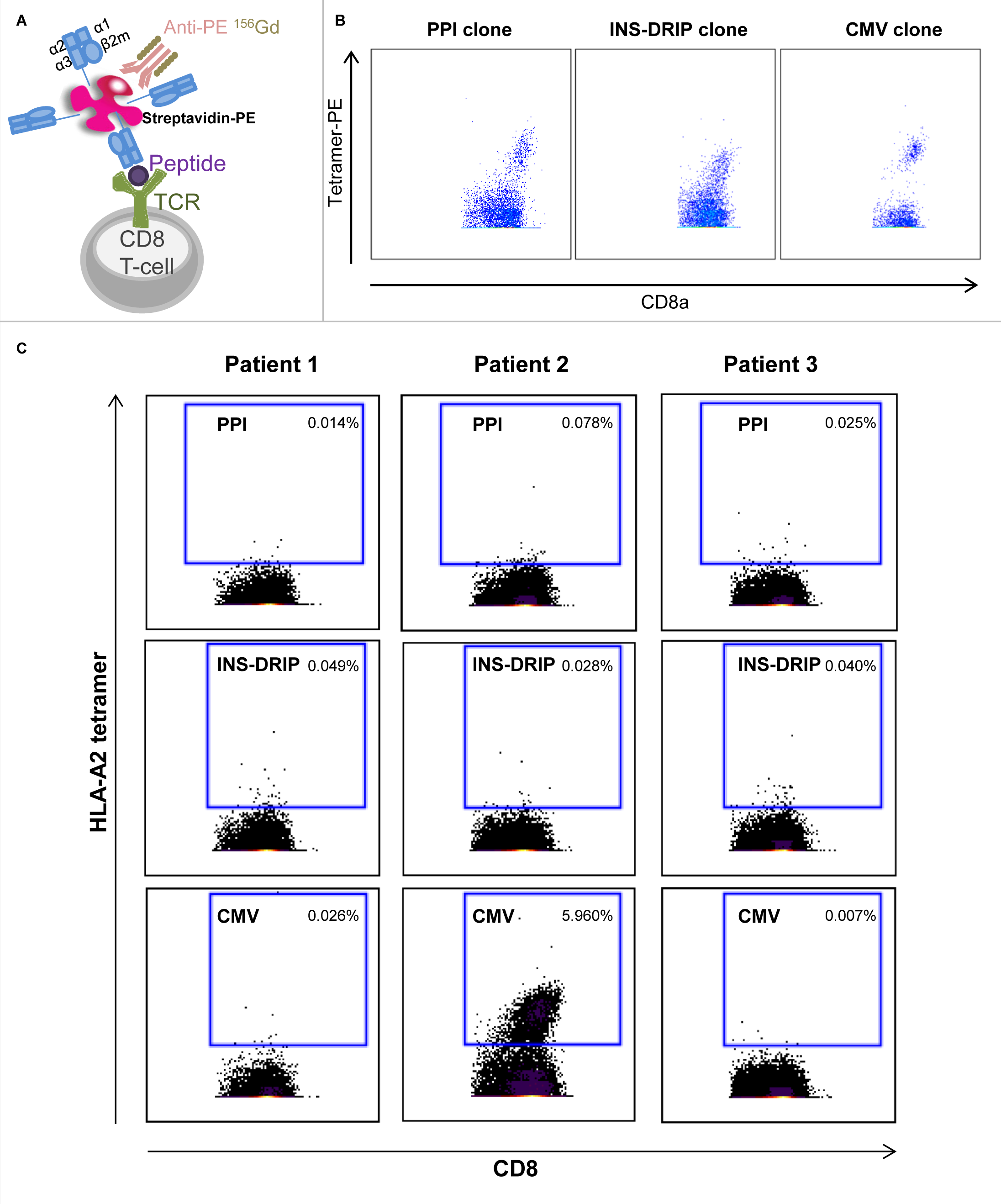

Supplement: S1 Fig — a) Schematic overview of staining peptide-specific CD8 T-cells using HLA-class I tetramers in CyTOF2. b) HLA-A2 negative PBMCs spiked with 1% PPI-specific (panel 1), 1% INS-DRIP-specific (panel 2) and 1% CMV-specific CD8 clone (panel 3). c) Dot plots and frequencies of circulating PPI, INS-DRIP and CMV reactive CD8 T-cells detected in three T1D patients. (TIF) [file pone.0200818.s001.tif]

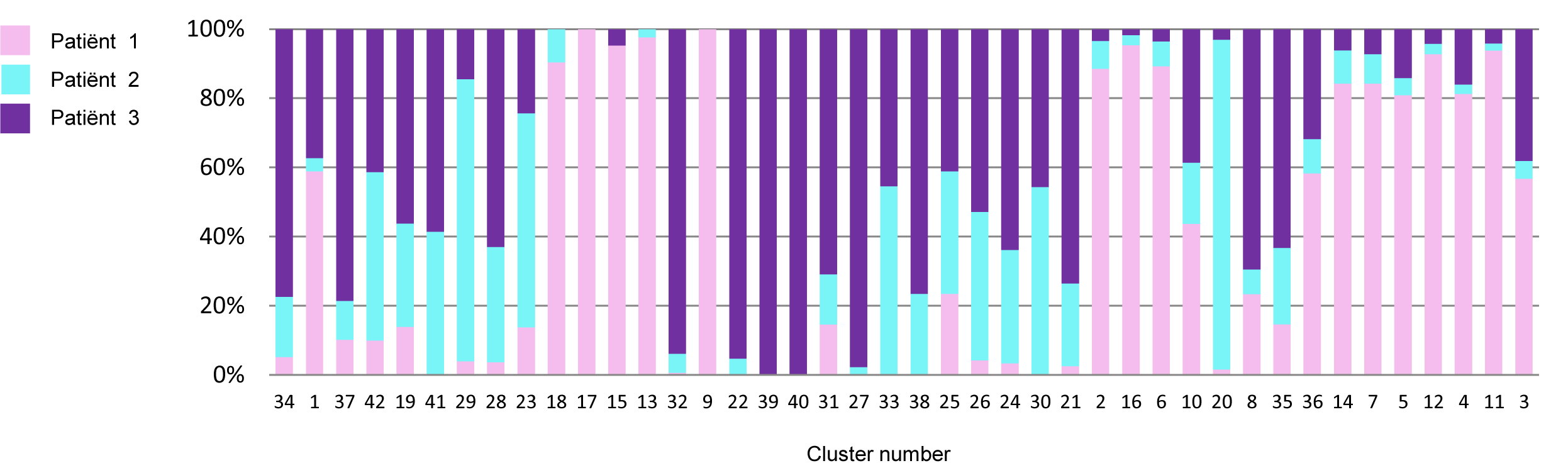

Supplement: S2 Fig — The graph shows a heterogeneous distribution of CD8 T-cells from different patients within the clusters. The order of the clusters corresponds to the heat map in Fig 1D. (TIF) [file pone.0200818.s002.tif]

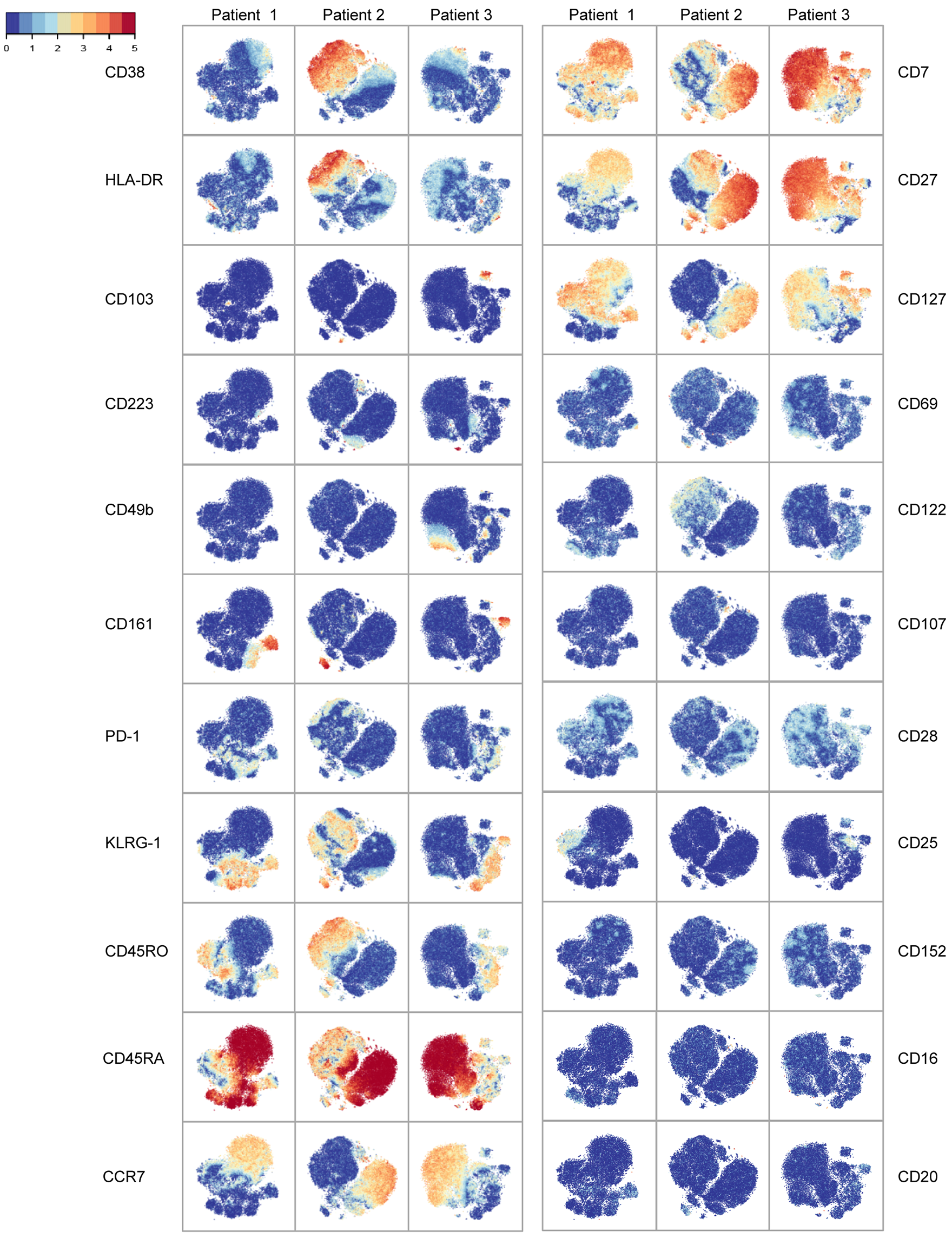

Supplement: S3 Fig — t-SNE embedding per patient, the marker expression per patient is displayed. Markers CD8, CD3 and TCRgd used for gating, as well as the negative markers NKp44 and NKp46 are not displayed. (TIF) [file pone.0200818.s003.tif]

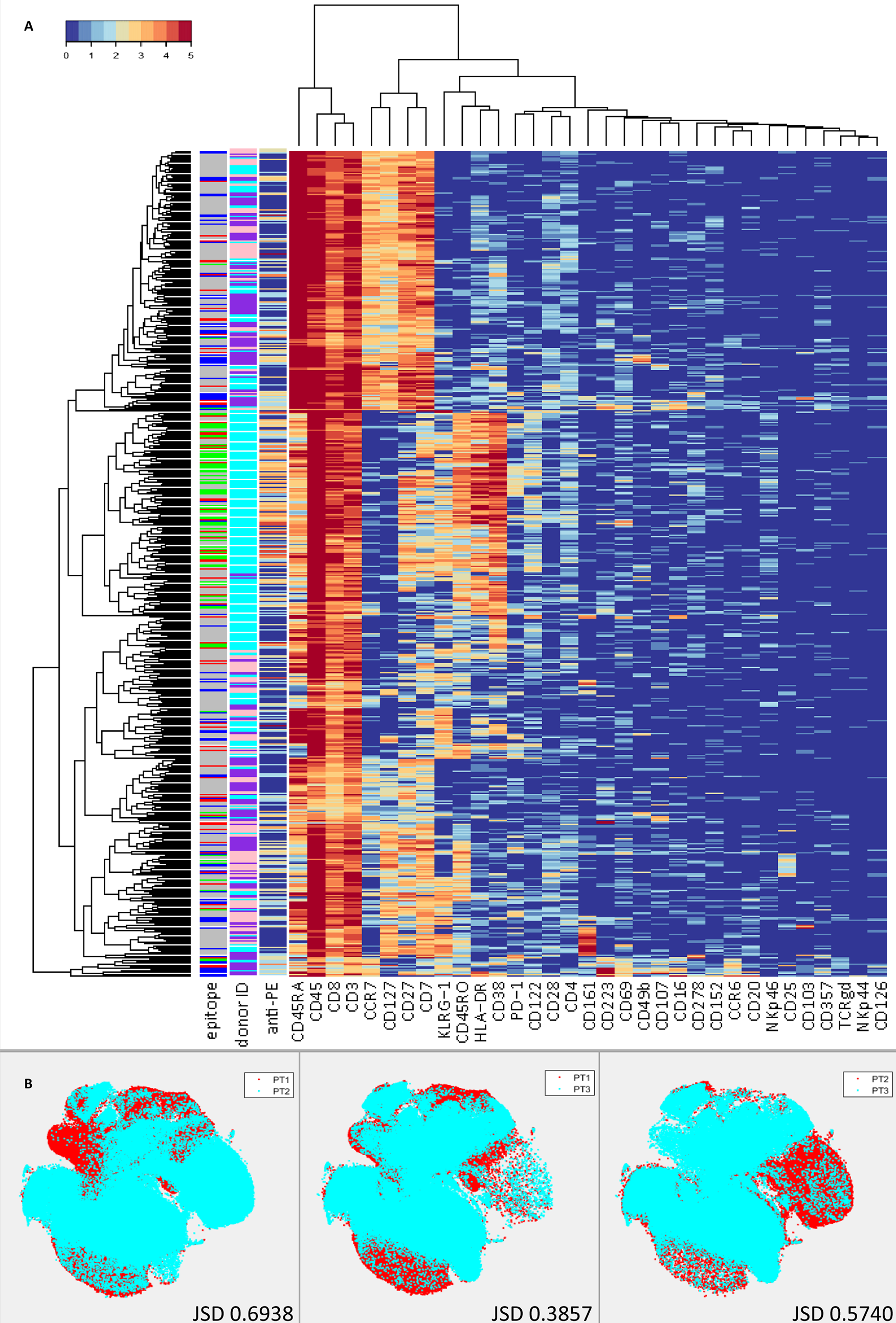

Supplement: S4 Fig — Left sidebar displays specificity (red = PPI, blue = INS-Drip, green = CMV, grey = CD8 tetramer negative, middle sidebar displays patient origin (pink = patient 1, light blue = patient 2, purple = patient 3), right sidebar displays the tetramer expression (anti-PE signal). b) t-SNE maps and Jensen-Shannon divergences values calculated in Matlab using SDivergenceTwoMaps. (TIF) [file pone.0200818.s004.tif]
