# Supplementary material for: Heterogeneity of circulating CD8 T-cells specific to islet, neo-antigen and virus in patients with type 1 diabetes mellitus
Source: PLoS One. 2018 Aug 8;13(8):e0200818. doi: 10.1371/journal.pone.0200818 (PMC6082515; doi:10.1371/journal.pone.0200818)
Supplement: S1 Table — (DOCX) [file pone.0200818.s005.docx]

**S1 Table. Information of patients diagnosed with Type 1 Diabetes Mellitus**.

| **Patient characteristics** | Patient 1 | Patient 2 | Patient 3 |
| --- | --- | --- | --- |
| Age, years | 43 | 23 | 34 |
| Gender | male | female | male |
| Onset age, years | 42 | 1 | 15 |
| Diabetes duration, years | 1 | 22 | 19 |
