# Supplementary material for: Heterogeneity of circulating CD8 T-cells specific to islet, neo-antigen and virus in patients with type 1 diabetes mellitus
Source: PLoS One. 2018 Aug 8;13(8):e0200818. doi: 10.1371/journal.pone.0200818 (PMC6082515; doi:10.1371/journal.pone.0200818)
Supplement: S2 Table — (DOCX) [file pone.0200818.s006.docx]

**S2 Table. Total number of events acquired per patient.**

|  | **PPI sample** | **INS-DRIP sample** | **CMV sample** |
| --- | --- | --- | --- |
| **Patient 1** | 464462 | 473368 | 225288 |
| **Patient 2** | 283817 | 405184 | 270707 |
| **Patient 3** | 500219 | 593990 | 595500 |
